# Practical Notes and Formulæ

**Published:** 1885-07-20

**Authors:** 


**Coffee Extract.**—

| | |
|---|---|
| Best Java coffee, (finely ground) | 4 ounces |
| Best Mocha coffee, (finely ground) | 4 ounces |
| Boiling water | 1 pint. |

This should stand one hour in a covered vessel, then be transferred to a percolator, and one pint of extract obtained by finishing the percolation with the *cold* menstruum, as given for tea. One ounce of the extract to a quart of light-weight syrup, say 22° to 25°, is sufficient.

An elegant method of dispensing the tea and coffee is, to put a small quantity of the extract in a cup or mug, drop in a block of sugar, or add a small quantity of cream syrup (if cream is called for), and draw the hot water on it.—*Pop. S. News.*

**Clymer's Mixture for Asthma.**—L'Union Medicale gives this anti-asthmatic remedy, suggested by Clymer:

| | | |
|---|---|---|
| ℞. | Tincture of opium | 4 grains |
| | Sulphuric ether | 8 grains |

Take fifty drops of this mixture every twenty minutes during the attack. Fifteen drops of the etheral tincture of lobelia may be added to each dose.—*Pop. S. News.*

**A Mixture for Whooping Cough.**—Roger ("Union Med.") is credited with this mixture:

| | | |
|---|---|---|
| ℞. | Syrup of belladonna | 1½ ounce |
| | Syrup of valerian | 6 drachms each. |
| | Syrup of digitalis | |

Each teaspoonful contains about one-twelfth of a grain of the extract of belladonna. The dose, for children under two years of age, is half a teaspoonful during the day, gradually increased every two days until two teaspoonfuls are administered in twenty-four hours. With children from two to five years of age the dose may be carried up to six teaspoonfuls daily. The mixture is particularly recommended during the second stage of the disease.—*New York Med. Journal.*

**Lyon's Kathairon.**—The following preparation is said to closely resemble Lyon's Kathairon for the hair:

| | | | |
|---|---|---|---|
| ℞. | Castor oil | cong. | 2 |
| | Alcohol | cong. | 3 |
| | Tinct. cantharides | fl. ℥. | 10 |
| | Oil of bergamot | fl. ℥. | 12 |
| | Tinct. red saunders | q. s. | |

Disolve the oil in the alcohol, add the tincture of cantharides, then the oil of bergamot, previously dissolved in a little alcohol. Make the tincture of red saunders in the proportion of 1 pound of the drug to 5 gallons of ninety-five per cent. alcohol; use 4 fluid ounces of this to each 30 gallons of the preparation.—ED. *National Druggist.*

**Sedative Cough Mixture.**—Dr. H. C. Wood, of the Therapeutic Gazette, has tested the following, and regards it as the most efficient sedative cough-mixture he has ever used :

℞. Potassii citratis .............................. ℥ j,
Succus limonis .............................. ℥ ij,
Syrup ipecac .............................. ℥ ss,
Syrup simplicem, q. s. ad .............................. ℥ vj.
M. Sig. : A tablespoonful from four to six times a day.

When there is much cough or irritability of the bowels he adds paregoric in suitable quantity.—*Medical Age.*

**Lotions for Pruritus of the Genitals.**—A contributor to the Union medicale credits Doyon with the following prescriptions :

℞. Bichloride of mercury } each ............. 4 grains,

Chloride of ammonium }

Almond mixture .............................. 13 ounces.

In case this fails, use :

℞. Chloral hydrate .............................. 75 grains,
Rose-water .............................. 3 ounces,
Distilled water .............................. 4½ ounces.

After the parts have been bathed with the lotion, they are to be powdered with starch.—*N. Y. Med. Journal.*

**Cocaine in the Treatment of Sore Nipples.** — Herrgott (Ann. de gynec. ; Union med.) sums up his experience as follows : 1. All the women with sore nipples who came under observation were able to give suck without pain after a 4-per cent. solution of cocaine hydrochlorate had been applied to the nipple. 2. The condition of the nipple was improved, and, where the cracks were not deep, they disappeared rapidly. 3. Cocaine should be used whenever the nipples are sensitive, in order to prevent fissures, the latter being often due to a shrinking movement on the part of the mother whenever the child seizes the breast.—*Ib.*

**Compound Carbolic Ointment.**—Mr. Thomas J. Covell, Hospital Steward at the National Home for disabled Volunteer Soldiers, Togus, Me., sends this formula :

℞. Carbolic acid (crystal) .............................. 1 ounce,
Balsam fir .............................. 2 ounces,
Yellow wax .............................. 4 ounces,
Deodoroleine or petrolatum .............................. 13 ounces.
M. ft. ung. s. a.

This gives an ointment that adheres, and is very serviceable in the treatment of old, chronic sores. I devised it for our surgeon, who wished one that would stick.—*Drug. Circular.*

**Glycerinum Aluminis.**—In the British Medical Journal for January 24, Mr. R. W. Parker proposes a preparation made by dissolving 1 ounce of alum in 5 ounces of glycerin, by aid of a gentle heat. While equal to tannin in astringency, it is superior in that it is less disagreeable, and is compatible with iron salts. Diluted, it may be used as a spray or gargle, lotion, or infection.—*Ib.*

